# DNA damage by lipid peroxidation products: implications in cancer, inflammation and autoimmunity

**DOI:** 10.3934/genet.2017.2.103

**Published:** 2017-04-18

**Authors:** Fabrizio Gentile, Alessia Arcaro, Stefania Pizzimenti, Martina Daga, Giovanni Paolo Cetrangolo, Chiara Dianzani, Alessio Lepore, Maria Graf, Paul R. J. Ames, Giuseppina Barrera

**Affiliations:** 1 Department of Medicine and Health Sciences “V. Tiberio”, University of Molise, Campobasso, Italy; 2 Department of Clinical and Biological Sciences, University of Torino, Torino, Italy; 3 Department of Drug Science and Technology, University of Torino, Torino, Italy; 4 Department of Molecular Medicine and Medical Biotechnologies, University of Naples Federico II, Naples, Italy; 5 CEDOC, NOVA Medical School, Universidade NOVA de Lisboa, Lisboa, Portugal, and Department of Haematology, Dumfries Royal Infirmary, Dumfries, Scotland, UK

**Keywords:** lipid peroxidation, aldehydes, DNA adducts, cancer, inflammation, autoimmunity

## Abstract

Oxidative stress and lipid peroxidation (LPO) induced by inflammation, excess metal storage and excess caloric intake cause generalized DNA damage, producing genotoxic and mutagenic effects. The consequent deregulation of cell homeostasis is implicated in the pathogenesis of a number of malignancies and degenerative diseases. Reactive aldehydes produced by LPO, such as malondialdehyde, acrolein, crotonaldehyde and 4-hydroxy-2-nonenal, react with DNA bases, generating promutagenic exocyclic DNA adducts, which likely contribute to the mutagenic and carcinogenic effects associated with oxidative stress-induced LPO. However, reactive aldehydes, when added to tumor cells, can exert an anticancerous effect. They act, analogously to other chemotherapeutic drugs, by forming DNA adducts and, in this way, they drive the tumor cells toward apoptosis. The aldehyde-DNA adducts, which can be observed during inflammation, play an important role by inducing epigenetic changes which, in turn, can modulate the inflammatory process.

The pathogenic role of the adducts formed by the products of LPO with biological macromolecules in the breaking of immunological tolerance to self antigens and in the development of autoimmunity has been supported by a wealth of evidence. The instrumental role of the adducts of reactive LPO products with self protein antigens in the sensitization of autoreactive cells to the respective unmodified proteins and in the intermolecular spreading of the autoimmune responses to aldehyde-modified and native DNA is well documented. In contrast, further investigation is required in order to establish whether the formation of adducts of LPO products with DNA might incite substantial immune responsivity and might be instrumental for the spreading of the immunological responses from aldehyde-modified DNA to native DNA and similarly modified, unmodified and/or structurally analogous self protein antigens, thus leading to autoimmunity.

## 1. Introduction

In recent years, it has become evident that lipid peroxidation (LPO) products are involved in the intracellular signaling mechanisms that determine the cell's final fate [1]. LPO arises from the oxidation of fatty acids induced by oxidative stress causing agents, e.g., oxidants, heat shock, UV and X irradiation, metal storage, excess caloric intake and serum starvation. Oxidative stress imports increases of reactive oxygen species (ROS) which, in turn, can affect signaling mechanisms in a concentration-dependent manner [2]. However, although increased ROS production has been observed in several human diseases, such as cancer and neurodegenerative diseases, an increase of LPO products is not always present. This is true in particular for cancer cells, which often display high levels of oxidative stress, whereas increased levels of LPO products were present only in some cancer types, depending on the lipid composition of cellular membranes, the presence of inflammation and the level of aldehyde metabolizing enzymes [3],[4]. On the contrary, in inflammatory and neurodegenerative diseases the increases of ROS almost always were accompanied by increases of LPO and, as a consequence, LPO products. Several studies have been performed regarding the biological roles played by aldehydes, since they have a prolonged half-life, can diffuse from their sites of formation and react with the surrounding cells. Moreover, the aldehydes can be delivered by the bloodstream and secreted in the urine. To the contrary, free radicals, produced during LPO, have a very short life and can produce only localized effects. For these reasons, the aldehydes have been defined as “second messengers of oxidative stress” [5]. These lipid electrophiles have long been studied, due to their potential to react with nucleophilic functional groups in lipids, proteins, and DNA [6]. The nucleophilic functional groups include sulfhydryl, guanidine, imidazole and amino groups and DNA bases. In particular, the aldehydes often attack the free -NH_2−_ groups of DNA bases to form covalent adducts, which are partially responsible for the biological consequences of LPO in normal physiology and pathophysiology. In this review we summarize the most recent evidence of DNA damage by LPO products in several diseases, such as cancer, inflammation and autoimmunity.

## 2. Aldehydes generated from lipid peroxidation

The first determination of aldehydes formed during LPO was provided by Esterbauer and Zollner [7]. These authors described three steps in the LPO process: initiation, propagation and termination. Initiation takes place by the free-radical, non-enzymatic peroxidation of lipids, which imports the abduction of a H· radical by a radical-initiating species (e.g., the hydroxyl radical ·OH) from a lipid, to yield a lipid radical; the formation of lipid radicals from polyunsaturated fatty acids (PUFAs) is favored, as they are resonance stabilized. Once formed, a lipid radical can react with oxygen to give a lipoperoxyl radical (LOO·) and a lipid hydroperoxide (LOOH). The unstable LOOHs generate new LOO· and alkoxyl (LO·) radicals, which can function as initiating species for new cycles of LPO and decompose into a complex mixture of more stable compounds, such as pyrroles, hydroxyoctadecanoic acids and aldehydes, the end products of LPO [8],[9]. Aldehydes have received much attention, because they are relatively stable, reactive and toxic [10],[11]. Kaway et al., by using gas chromatography/electron ionization/mass spectrometry with a selected ion monitoring system, detected several products of arachidonic acid, linoleic acid, and docosahexaenoic acid peroxidation [12]. These authors identified 33 different aldehydes, which were classified into five groups: alkanals, 2-alkenals, 2,4-alkadienals, 2-hydroxyalkanals, 4-hydroxy-2-alkenals, and three other compounds, which were not comprised in the previous groups: glyoxal, malondialdehyde, and 4,5-epoxy-2-decenal. From a quantitative standpoint, the major aldehyde products are malondialdehyde (MDA), acrolein, 4-hydroxy-2-nonenal (HNE), and 4-oxo-2(*E*)-nonenal (ONE).

**Figure 1.**
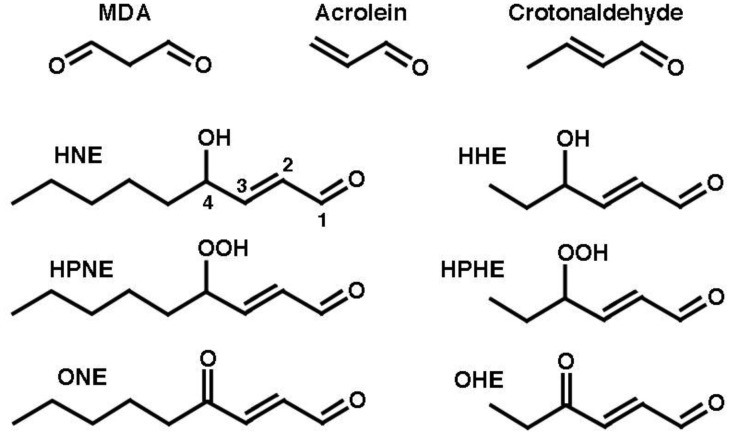
Representative aldehydes produced in the course of LPO. MDA, malondialdehyde; HNE, 4-hydroxy-2-nonenal; HHE, 4-hydroxy-2-heptenal; HPNE, 4-hydroperoxy-2-nonenal; HPHE, 4-hydroperoxy-2-heptenal; ONE, 4-oxo-2-nonenal; OHE, 4-oxo-2-heptenal.

### 2.1. MDA

MDA can be produced by the peroxidation of PUFAs which contain more than two C-C double bonds, such as 4,7,10,13,16,19-docosahexaenoic acid (22:6[*n*-3], DHA) and 5,8,11,14-eicosatetraenoic acid (20:4[*n*-6], arachidonic acid, AA). As these represent the majority of PUFAs, MDA is the major product of LPO. The peroxidation of *n*-3 (ω-3)-PUFAs, such as 9,12,15-octadecatrienoic acid (18:3[*n*-3], alpha-linolenic acid, ALA) and DHA, can generate 4-hydroperoxy-2(*E*)-hexenal (HPHE) and, thenceforth, 4-hydroxy-2(*E*)-hexenal (HHE). On the other hand, the peroxidation of *n*-6 (ω-6)-PUFAs, like 9,12-octadecadienoic acid, (18:2[*n*-6], linoleic acid, LA) and AA, can yield 4-hydroperoxy-2(*E*)-nonenal (HPNE), and HNE, while 4-hydroxyundecenal arises from ω-9 PUFAs [11]. Because ω-6 PUFAs are most abundant, the level of HNE formed largely exceeds those of HHE and 4-hydroxyundecenal [13]. Figure 1 shows the structures of MDA, acrolein and some representative 4-substituted 2-alkenals.

### 2.2. 4-Hydroxy-alkenals

4-Hydroxy-alkenals, such as HHE and HNE, have three chemical functions (the aldehyde group, the C2-C3 double bond and the OH group at chiral centre C4), which make them highly reactive.

**Figure 2.**
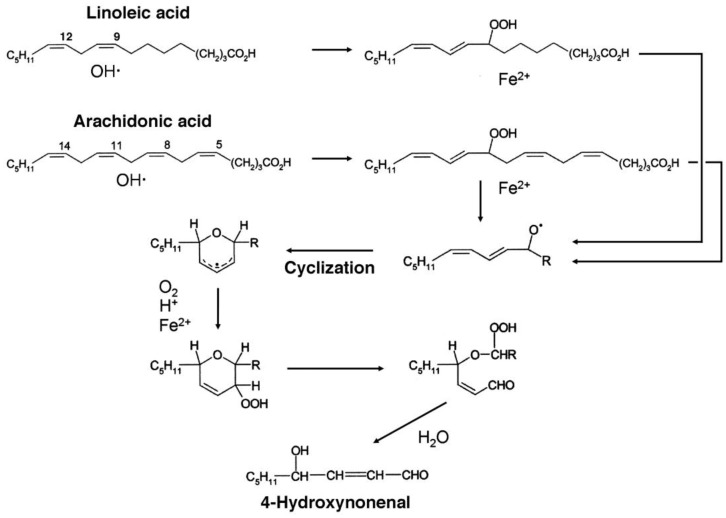
Mechanism of HNE formation by the peroxidation of arachidonic acid.

The mechanism of formation of HNE via 9-hydroperoxy-linoleic acid or 11-hydroperoxy-arachidonic acid is depicted in Figure 2. Notice that the positional isomers of the latter two compounds, 13-hydroperoxy-linoleic acid and 15-hydroperoxy-arachidonic acid, respectively, are also admitted as possible precursors of HNE [14].

HNE has been identified as a result of auto-oxidation and LPO in liver microsomes [15]. The amount of HNE formed is up to 80 fold lower than that of MDA [16], but MDA is much less reactive than HNE, ONE or acrolein [17]. The amount of HNE within cells is dependent not only on the rate of formation during LPO but also on its metabolism, which is regulated by enzymes such as aldose reductase (AR), glutathione-S-transferases (GSTs), aldehyde dehydrogenases (ALDHs), and alcohol dehydrogenases (ADHs) [17]. The production of HNE has well proven implications in the pathogenesis of human cancer, neurodegenerative and chronic inflammatory diseases, such as atherosclerosis [18].

**Figure 3.**
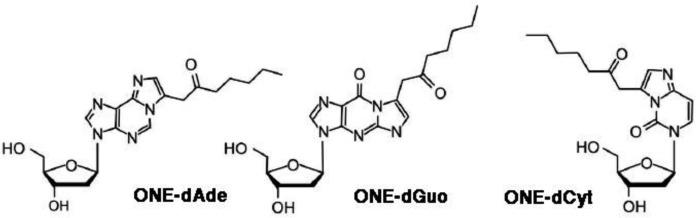
Structures of the 2″-oxo-heptyl-substituted 1,*N*2-etheno-2′-deoxyguanosine, 1,*N*6-etheno-2′-deoxyadenosine and 3,*N*4-etheno-2′-deoxycytidine ONE adducts.

4-oxo-2-nonenal (ONE) is the main product of decomposition *in vitro* of both (S)-regioisomers of linolenic acid hydroperoxide, i.e., 13(*S*)-hydroperoxy-9,11-octadecadienoic acid (13(*S*)-HPODE), and 9(*S*)-HPODE [19]. ONE is more reactive than HNE, and reacts in different ways with various biomolecules [20]. In particular, ONE forms 2″-oxo-heptyl-substituted 1,*N*2-etheno-2′-deoxyguanosine [21], 1,*N*6-etheno-2′-deoxyadenosine [21],[22] and 3,*N*4-etheno-2′-deoxycytidine [23] (Figure 3). Etheno-type adducts with DNA are formed also by 4-oxo-2(*E*)-hexenal (OHE) [24].

### 2.3. Acrolein

Acrolein is generated by the oxidation of unsaturated lipids, but unlike the other aldehydes, the majority of acrolein found *in vivo* derived from environmental exposure [25]. Moreover, acrolein is also generated by the degradation of polyamines or by myeloperoxidase present in neutrophils [26].

## 3. Reactions of LPO-derived aldehydes with DNA and functional consequences

### 3.1. MDA

**Figure 4.**
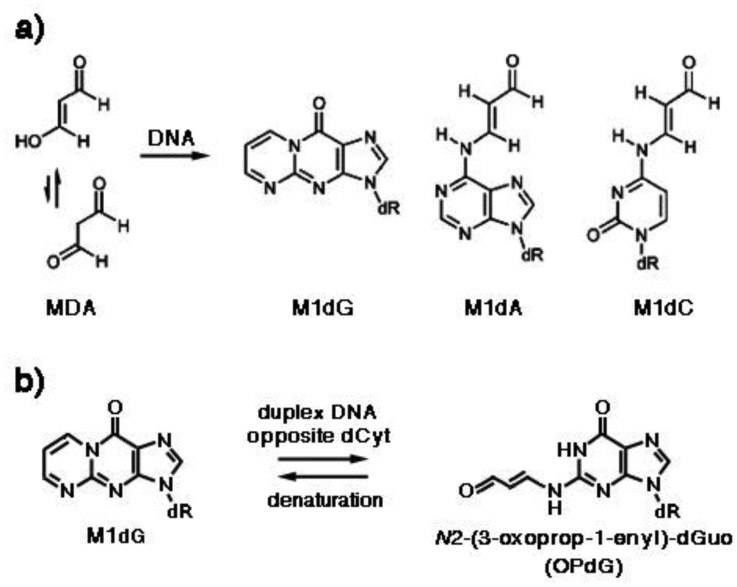
(a) Adducts of MDA with deoxyguanosine, deoxyadenosine and deoxycytidine. M1dG, pyrimido[1,2-α]purine-10(3*H*)-one-2′-deoxyribose; M1dA, *N*6-(3-oxoprenyl)deoxyadenosine; M1dC, *N*4-(3-oxoprenyl)deoxycytidine. (b) Reorganization of M1dG when placed opposite dCyt in duplex DNA. OPdG, *N*2-(3-oxoprop-1-enyl)-deoxyguanosine.

MDA is mutagenic both in bacteria [27] and in human cells, causing insertions, deletions and base pair substitutions, particularly at GC base pairs [28]. Both MDA carbonyls react with nitrogen, forming the pyrimido[1,2-α]purine-10(3*H*)-one-2′-deoxyribose, or malondialdehyde-2′-deoxyguanosine adduct (M1dG), which is the most abundant MDA adduct, while deoxyadenosine and deoxycytidine adducts arise from the addition of one carbonyl with exocyclic amino groups to form *N*6-(3-oxoprenyl)deoxyadenosine (M1dA) and *N*4-(3-oxoprenyl)deoxycytidine (M1dC), respectively (Figure 4a). The former yields about 20% of M_1_G, whereas M_1_C is formed only in trace amounts [29]. M_1_G adducts are detected in tissues from healthy humans [30],[31], are mutagenic in bacteria and susceptible to nucleotide excision repair. Mutations induced by M1dG, using MDA-modified M13 genomes replicated in *E. coli*, included M1dG → A, M1dG → T, and low levels of M1dG → C mutations. However, the mutation frequency was only 1% when deoxycytidine (dCyt) was placed opposite the lesion [32]. It was observed that M1dG placed opposite dCyt underwent a configurational rearrangement with opening of the exocyclic ring to *N*2-(3-oxoprop-1-enyl)-deoxyguanosine (OPdG) (Figure 4b), via a second-order reaction with hydroxide catalyzed by the complementary dCyt, whose reversal in acid was very slow [33]. The OPdG propenyl chain is located in the minor DNA groove, thus facilitating *Watson-Crick* H-bonding with dCyt [34], which may explain why M1dG is weakly mutagenic [35].

### 3.2. Acrolein

**Figure 5.**
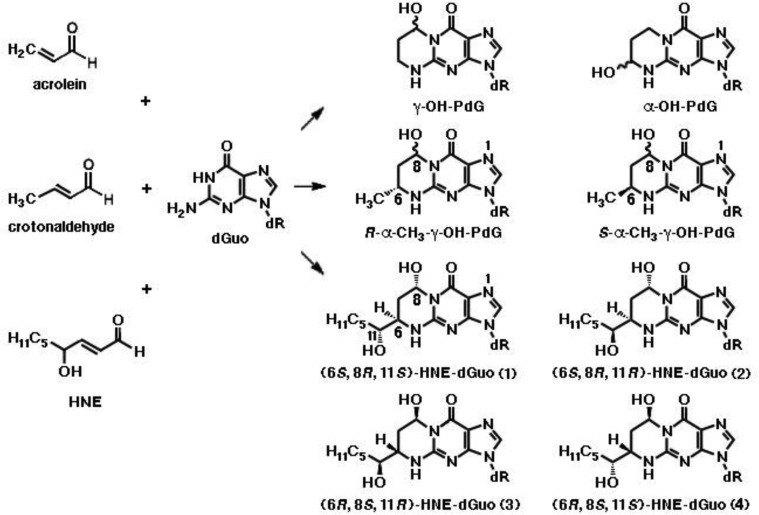
1,*N*2-exocyclic propane adducts generated by reaction of acrolein, crotonaldehyde and HNE with deoxyguanosine (dGuo) in DNA. γ-OH-PdG, acrolein-derived γ-hydroxy-1,*N*2-propano-2′-deoxyguanosine adduct; α-OH-PdG, α-hydroxy-1,*N*2-propano-2′-deoxyguanosine adduct of acrolein; α-CH_3_-γ-OH-PdG, α-methyl-γ-hydroxy-1,*N*2-propano-2′-deoxyguanosine adduct of crotonaldehyde. Four diastereomers of the latter are formed, among which those with the *trans* configuration of γ-OH and α-CH3 predominate, with the major and minor epimers at C(8) interconverting in single-strand DNA. Also, four diastereomers (1-4) of the 1,*N*2-propano-2′-deoxyguanosine adduct of HNE (HNE-dGuo) exist, as a result of the *trans* configuration between the alkyl side chain at C(6) and the hydroxyl group at C(8) and the presence of chiral C(11) in the alkyl chain [48].

**Figure 6.**
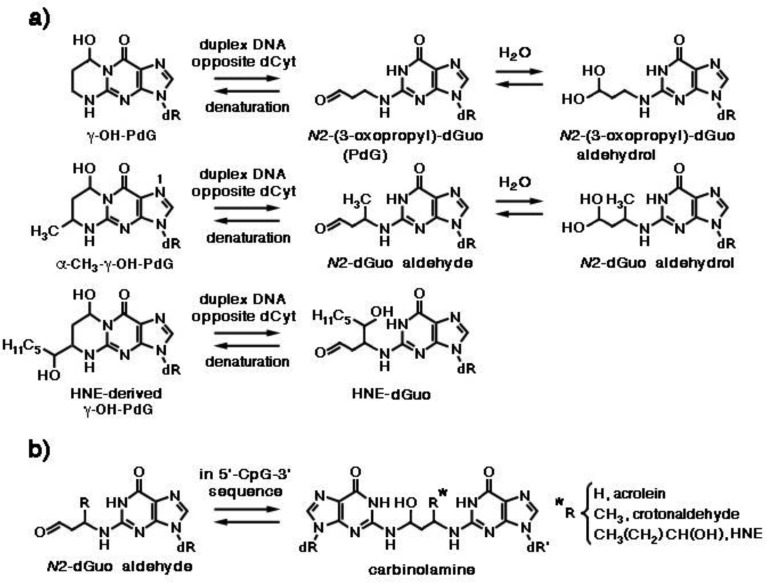
(a) Reorganization of *N*2-propane adducts of acrolein, crotonaldehyde and HNE with deoxyguanosine when placed opposite dCyt in duplex DNA. PdG, *N*2-(3-oxopropyl)-deoxyguanosine. (b) Formation of *N*2-dGuo:*N*2-dGuo cross-links.

Acrolein displays mutagenic properties in bacteria and mammalian cells [27], as well as carcinogenic properties in rats [36]. It is a major cigarette smoke-related lung carcinogen, as the pattern of formation of acrolein-DNA adducts closely parallels the mutational pattern of the *p53* gene in human lung cancer cells [37]. Acrolein-derived adducts have been detected in human and rodent DNA [38]–[41]. Michael addition of acrolein to the *N*2-amino group of deoxyguanosine, followed by ring closure, results in the formation of γ-hydroxy-1,*N*2-propano-2′-deoxyguanosine (γ-OH-PdG) and α-hydroxy-1,*N*2-propano-2′-deoxyguanosine (α-OH-PdG) (Figure 5). DNA replication across γ-OH-PdG occurs correctly in bacteria and mammalian cells [42],[43]. Like M1dG, γ-OH-PdG also undergoes ring opening when placed opposite dCyt, forming *N*2-(3-oxopropyl)-deoxyguanosine (PdG) (Figure 6a) [44], which facilitates canonical *Watson-Crick* H-bonding with the complementary dCyt. This may explain the weak mutagenic properties of γ-OH-PdG [35]. Yeast Rev1 DNA polymerase incorporates the correct nucleotide dCyt opposite PdG, a model for γ-OH-PdG, with similar efficiency as if it were opposite an undamaged dGuo, but further extension of the polynucleotide requires the Pol ζ polimerase [45]. The crystal structure of the Rev1 polymerase in complex with PdG-modified DNA revealed a conformational interconversion of the *N*-glycosyl bond connecting the deoxyribose sugar to the nucleobase. The latter shifts from the *anti* conformation, (in which it is oriented away from deoxyribose and *Watson-Crick* H-bonding interactions between complementary nucleobases are permitted), to the *syn* conformation, which positions PdG into a small hydrophobic cavity, with incoming dCTP interacting with an Arg residue via two H-bonds [45]. In contrast, α-OH-PdG is stable when placed opposite dCyt and blocks replication in human cells [43]. It adopts a *syn* conformation around the glycosyl bond, forming a non-mutagenic *Hoogsteen* pair to its complementary dCyt [46]. Rev1 and Pol τ polymerases mediate accurate replication across α-OH-PdG, with the latter incorporating dATP and dTTP at low frequencies [47].

### 3.3. Crotonaldehyde

Addition of crotonaldehyde to dGuo produces four diastereomers of α-CH_3_-γ-OH-PdG, as a result of the presence of chiral C(6), among which those with the *trans* configuration of γ-OH and α-CH_3_ predominate, with the major and minor epimers at C(8) interconverting in single-strand DNA (Figure 5). When the crotonaldehyde-derived γ-OH-PdG is placed opposite deoxythymidine in double-strand DNA, the ring-opened species is undetectable, like in single-strand DNA. However, when placed opposite dCyt, both *(R)*- and *(S)*-α-CH_3_-γ-OH-PdG adducts undergo incomplete ring opening to *N*2-dGuo aldehydes and respective *N*2-dGuo aldehydrols (Figure 6a) [49].

### 3.4. HNE

Genotoxic effects of HNE, such as DNA fragmentation and sister chromatide exchange (SCE), were first detected in CHO cells [50], whereas in V79 Chinese hamster cells mutations of the *HGPRT* gene were induced by HNE in a dose-dependent manner [51]. Primary hepatocytes proved to be most sensitive to the genotoxic effects of LPO-derived aldehydes, with HNE exhibiting higher SCE-inducing potential than both its analogues lacking either the OH group (2-*trans*-nonenal) or the OH and the CC double bond (nonanal), and aldehydes with lipophilic tails of different lengths, such as 4-hydroxyhexenal and 4-hydroxyundecenal [52]. Significant levels of SCE occurred at HNE concentrations as low as 0.1 μM, like those that can be attained under physiological conditions [53]. Genotoxic effects of HNE, such as micronuclei and chromosomal aberrations, were observed also in brain endothelial cells at concentrations ≥1 μM [54].

Various patterns of HNE interaction with DNA have been described [5]. Michael addition of the *N*2-amino group of deoxyguanosine (dGuo) to HNE, followed by ring closure of N1 onto the aldehyde, results in four diastereomeric cyclic γ-hydroxy-1,*N*2-propano-2′-deoxyguanosine (γ-OH-PdG) adducts (Figure 5) [48]. These were detected as endogenous lesions in tissues of untreated rats and humans, particularly in the liver, and their levels greatly increased in liver DNA of F344 rats treated with CCl4, indicating LPO as their probable source [48],[55]. In cultured human monocytes incubated with HNE, the 1,*N*2-propano-2′-deoxyguanosine adduct of HNE (HNE-dGuo) largely predominated, with respect to the MDA-2′-dGuo adduct (M1G) cited above and the 1,*N*6-etheno-deoxyadenosine and 1,*N*2-etheno-deoxyguanosine adducts, with their 1″,2″-dihydroxyheptyl-substituted derivatives discussed below, all of which were detected in much lower yields [56]. It was suggested that they might have a pathogenetic role in hepatic carcinogenesis, as HNE preferentially formed adducts with DNA at codon 249 of the human *p53* gene, which is a mutational hotspot in hepatocellular carcinoma [57]. Site-specific mutagenesis studies revealed that the (6*S*,8*R*,11*S*)- and (6*R*,8*S*,11*R*)-1,*N*2-HNE-dGuo adducts are mutagenic, as they are able to induce low levels of G → T transversions and G → T transitions. Moreover, when 1,*N*2-HNE-dGuo adducts are placed opposite dCyt in duplex DNA, the exocyclic ring opens (Figure 6a), permitting the correct *Watson-Crick* base pairing for the adducted deoxyguanosine [58]. However, when mismatched with dAde in DNA, (6*S*,8*R*,11*S*)-1,*N*2-HNE-dGuo maintains its exocyclic ring, a situation mimicking the incorrect incorporation of dATP (G → T transversion) [59]. The adduct conformation is in equilibrium between the *syn-* and the *anti-* conformation around the glycosyl bond. In the *syn-* conformation, which is favored at acidic pH, the HNE moiety is located in the major groove, dAde is protonated and the (6*S*,8*R*,11*S*)-1,*N*2-HNE-dGuo (*syn*):dAde^+^ (*anti*) base pair is stabilized by *Hoogsteen* type H-bonding.

The 1,*N*2-dGuo enal adducts formed by acrolein, crotonaldehyde and HNE (Figure 5) are capable of forming interstrand *N*2-dGuo:*N*2-dGuo cross-links (Figure 6b). Interstrand cross-links represent one of the worst kinds of DNA damage, as they impede the separation of complementary strands, which is required both for DNA replication and transcription. The formation of interstrand cross-links can be induced by enals in the 5′-CpG-3′ sequence, but not in the 5′-GpC-3′ sequence [60]–[62] and requires the rearrangement of the γ-OH-PdG adducts to the ring-opened *N*2-dGuo aldehydes (Figure 6a). In duplex DNA, the *N*2-dGuo:*N*2-dGuo linkages are found predominantly as carbinolamines, with the carbinolamine linkage maintaining the canonical *Watson-Crick* base pairing [63],[64]. The HNE-derived γ-OH-PdG adduct is fully converted to cross-link [62] and is extremely stable, whereas less than 50% of the γ-OH-PdG adducts deriving from acrolein and crotonaldehyde are converted to cross-links [60],[61]. The configuration of the γ-OH-PdG adducts influences interstrand cross-linking. The crotonaldehyde-derived (*R*)-CH_3_-γ-OH-PdG adduct induces cross-linking more efficiently than the (*S*)-CH_3_-γ-OH-PdG adduct [61]. Of the four stereoisomers of HNE-derived γ-OH-PdG adducts, only the (6*S*,8*R*,11*S*)-configurated one induces interchain cross-linking [62].

**Figure 7.**
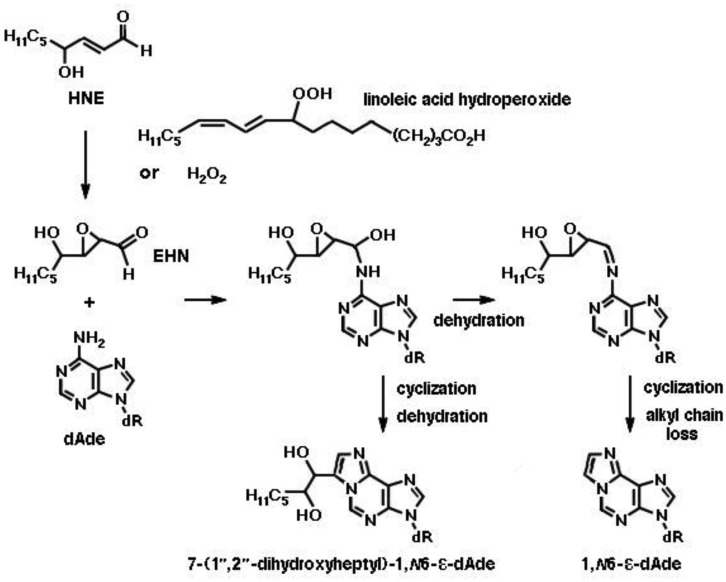
Exocyclic etheno adducts formed by the reaction of 2,3-epoxy-4-hydroxynonanal (epoxy-HNE, EHN) with deoxyadenosine (dAde) in DNA. EHN is formed from HNE by incubation in the presence of a fatty acid hydroperoxide (linoleic acid hydroperoxide in the example) or hydrogen peroxide at 37 °C for 24 h [65]. Upon addition of EHN at the exocyclic N6 amino group of dAde, an intermediate is formed, which may either undergo cyclization by ring closure at position N1 and dehydration, yielding 7-(1′,2′-dihydroxyheptyl)-1,*N*6-etheno-deoxyadenosine (7-(1′,2′-dihydroxyheptyl)-1,*N*6-ε-dAde), or dehydration into an imine, followed by cyclization and loss of the alkyl side chain by a retroaldol reaction, yielding 1,*N*6-etheno-deoxyadenosine (1,*N*6-ε-dAde).

**Figure 8.**
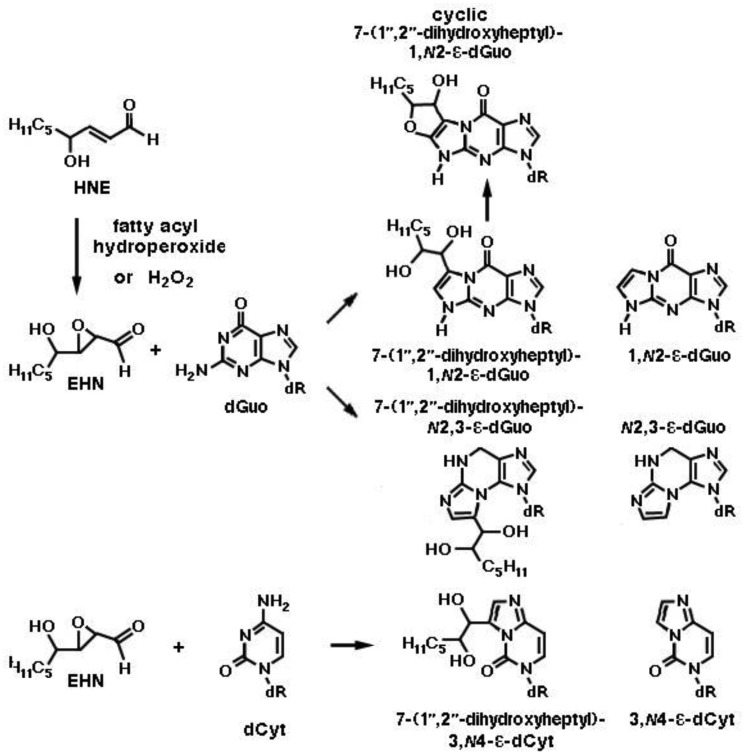
Exocyclic etheno adducts formed by the reaction of 2,3-epoxy-4-hydroxynonanal (epoxy-HNE, EHN) with deoxyguanosine (dGuo) and deoxycytidine (dCyt) in DNA. Two kinds of etheno-deoxyguanosine adducts, 1,*N*2-etheno-2′-deoxyguanosine (1,*N*2-ε-dGuo) and *N*2,3-etheno-2′-deoxyguanosine (*N*2,3-ε-dGuo), are presented. As with the reaction of EHN with dAde depicted in Figure 7, all three reactions shown here proceed via ethano intermediates (not shown), from which alkyl-substituted etheno adducts are formed by cyclization and dehydration, while unsubstituted etheno adducts are formed by base-catalyzed dehydration, cyclization and alkyl chain loss. Notice that two diastereomers exist of each of the 7-(1″,2″-dihydroheptyl)-substituted etheno adducts. In addition, a bicyclic derivative (cyclic 7-(1″,2″-dihydroheptyl)-1,*N*2-ε-dGuo), of which four diastereomers exist, is uniquely derived from the reaction of HEN with dGuo, by trapping of the cyclic imine by the side chain hydroxyl group. ε-dCyt, 3,*N*4-etheno-deoxycytidine.

Still more adducts are formed by reaction of mutagenic 2,3-epoxy-4-hydroxy-nonanal (EHN) with nucleobases. EHN is generated by incubation of HNE with fatty acid hydroperoxides (e.g., 9- or 13-linoleic acid hydroperoxide) or hydrogen peroxide at 37 °C for 24 h, in 13% and 21.5% yields, respectively [65]. It is more reactive towards DNA then the parent aldehyde HNE, forming 1,*N*6-etheno-deoxyadenosine and 1,*N*2-etheno-deoxyguanosine [66]. The formation of the adenosine etheno-bases from 2,3-epoxy-4-hydroxy-nonanal is depicted in Figure 7. Exocyclic etheno adducts formed upon reaction of 2,3-epoxy-4-hydroxy-nonanal with DNA include 1,*N*2-etheno-2′-deoxyguanosine, *N*2,3-etheno-2′-deoxyguanosine and 3,*N*4-etheno-deoxycytidine [38],[39], which are shown in Figure 8. In the liver, EHN may be formed by the action of cytochrome P-450 [65] and is not a substrate of epoxide hydrolase [67]. Both propano- and etheno-type adducts of HNE have been detected as endogenous lesions in the liver and other tissues of humans and rodents [38].

## 4. Aldehydes and DNA damage in cancer

### 4.1. Effects of aldehydes in inducing malignant transformation of normal cells.

DNA damage has been implicated in the development of cancer, and LPO products play a role in the induction of mutations responsible for DNA modifications which can induce carcinogenesis. As previously described, reactive oxidants can induce DNA base modifications and are a potential source of exocyclic adducts, most of which are mutagenic [6]. Although the main reactions of enals are with deoxyguanosine (dGuo) [68], Kowalczyk et al. demonstrated that all four DNA bases are targets for HNE, but they display different reactivities: dG > dC > dA ≈ dT [69]. As seen above, HNE is also able to form interchain cross-links in the 5′-CpG-3′ sequence (Figure 6b), which might interfere with DNA replication and transcription, thereby contributing to the etiology of human disease [70]. Moreover, both the G→T transversion at codon 249 of the *p53* gene and the HNE-dGuo adduct at the same codon have been considered a mutational hotspot in human hepatocellular carcinoma and in cigarette smoke-related lung cancer [57],[71],[72]. The formation of aldehyde-DNA adducts has been demonstrated *in vivo*, after LPO stimulation in Fisher rats by exposure to CCl_4_. Wacker et al. found a significant amount of HNE-dGuo adducts (>100 nmol/mol, a 37-fold increase) in rat liver, which was associated with a high incidence of liver cancer [55]. Further mechanistic insight into the pro-carcinogenic action of HNE comes from the demonstration that HNE increases the number of unrepaired single-strand breaks in cells treated with oxidizing or methylating agents, by increasing the rate of AP-site incision and blocking the re-ligation step after gap-filling by DNA polymerases, thus compromising the base excision repair pathway [73]. This inhibitory action on DNA repair has also been reported for acrolein, which represents a major lung and bladder carcinogen, and whose carcinogenic potential depends not only on the induction of DNA damage, but also on the inhibition of DNA repair [74].

MDA reacts with DNA to form adducts with deoxyguanosine and deoxyadenosine (Figure 4). The carcinogenic effect of MDA is supported by the detection of M1dG, the major adduct of MDA with DNA, in human tissues from larynx cancer [75] and breast cancer [76].

ONE is another well studied product of LPO, which can form alkylated etheno-DNA adducts (Figure 3). These were found in a colorectal cancer mouse model, together with increased cyclooxigenase -2 levels [77], and in samples of gastric mucosa from gastric cancer biopsies [78]. Finally, acrolein-DNA adducts were found in human bladder cancer [79] and in lung cancer of cigarette smokers [37].

Another aspect of the pro-carcinogenic effect displayed by aldehydes, and in particular by HNE, was highlighted by the observation that HNE may act as a proliferative factor in normal cell lines. In human hepatic stellate cells, HNE increased proliferation by targeting the p46 and p54 isoforms of c-Jun [80]. Moreover, in human B lymphocytes infected with EBV, a physiological concentration of HNE induced cell proliferation and latent membrane protein-1 (LMP1) expression [81]. Other data suggest that HNE can evoke signaling for defense mechanisms, including NF-E2-related factor 2 (Nrf2) signaling, thus self-regulating its own toxicity [82]. The stimulation of the Nrf2 pathway induces the expression of a wide variety of genes: antioxidant enzymes, including thioredoxine, thioredoxine reductase and heme-oxigenase, and enzymes related to the synthesis and conjugation of GSH, such as glutamate-cysteine ligase and glutathione-S-transferases (GSTs) [83]. Thus, the activation of this pathway can results in a more rapid extrusion or inactivation of electrophilic compounds, including aldehydes.

Since the production of ROS and the consequent induction of LPO can be involved in carcinogenesis, the use of antioxidants has been proposed as a potential therapeutic intervention, due to their ability to oppose oxidative stress in fibrosis and cancer development [84]. However, not all antioxidant supplements displayed protective effects against cancer development, as reported by Poljšak and Fink, in a comprehensive review [85].

### 4.2. Effects of aldehydes on cancer cells

It has been convincingly demonstrated that the alterations provoked by aldehydes can be responsible for carcinogenic effects in normal cells. However, in cancer cells the aldehydes can exert anticancerous effects as well. As it occurs in normal tissues, the production of LPO-derived aldehydes in cancer cells depends on the presence of reactive oxygen species (ROS). In recent years, it has become evident that many types of cancers, including hematological and solid tumors, produce large amounts of ROS, compared to their normal counterparts, due to an aberrant metabolism, mitochondrial dysfunction, the activation of oncogenes and the presence of inflammatory cells, such as granulocytes, which are a source of ROS [86]. The large amount of ROS can increase the formation of LPO products. An increased level of oxidatively damaged products, such as the oxidized DNA base 8-hydroxydeoxyguanosine (8-OHdG), has been demonstrated in solid tumors, including thyroid neoplasia [87], squamous cell carcinoma [88], non-small cell lung cancer [89], and prostate cancer [90]. This characteristic makes cancer cells more vulnerable to damage by further ROS production induced by exogenous agents [86]. In this context, ROS may exert a cytotoxic effect, leading to the death of malignant cells and thus limiting cancer progression [91].

However, while increases of oxidative stress have been demonstrated in the majority of cancer types, the concentration of LPO products in cancer cells may vary, in relation to several biological characteristics of tumor cells, such as the pattern of aldehyde metabolizing enzymes, the concentration of lipid peroxidable substrates, such as PUFAs, in cell membranes, and the presence of inflammatory cells, which can increase the level of diffusible aldehydes from the tumor-surrounding tissues [92]. Aldehyde metabolism is sustained by three major enzymes: alcohol dehydrogenases; aldehyde dehydrogenase; and glutathione-S-transferases (GSTs) [93], whose activity can differ, depending on the tumor type. For example, it has been shown that, during rat liver carcinogenesis, the activities of the enzymes metabolizing aldehydes was increased, thus rendering cancer cells more protected against the cytotoxic effect of aldehydes [4]. Moreover, tumor cells can export aldehyde-GSH conjugates, in an ATP-dependent manner or by the action of Ral-interacting protein (RLIP76), a GTPase-activating membrane protein, which has been found at increased levels in the majority of malignant tumors and is important for the acquisition of drug resistance [94],[95]. For example, in hepatoma cells, most HNE was converted to the HNE-GSH conjugate, which was rapidly and efficiently exported out of the cells [96].

The exogenous addition of LPO-derived aldehydes can affect cancer cell proliferation. The effects of HNE on cancer growth have been recently reviewed by Gasparovic et al. [97]. As reported, HNE seems to act as a biphasic factor: it stimulates proliferation at low doses and causes suppressive/cytotoxic effects at high doses [98]. Several reports have highlighted the effects of high HNE doses in inhibiting proliferation and inducing apoptosis of cancer cells. HNE inhibited the proliferation of human colon tumor cells, through regulation of the MAP kinases pathway [99], or through the PPAR gamma pathway [100]. In human leukemic cells, HNE inhibited c-myc [101], G1 cyclin expression [102] and telomerase activity [103]. Moreover, the inhibition of cell proliferation and the activation of p53 were also reported in breast cancer cells (MCF7) treated with conjugated linoleic acid (CLA), which increases the endogenous levels of HNE [104]. In human osteosarcoma cells treated with HNE, the induction of apoptosis was also evidenced [105]. In PC3 prostate carcinoma cells, HNE significantly potentiated the antitumor effects of the histone deacetylase (HDAC) inhibitor panobinostat (LBH589). Cell cycle analysis revealed that each of the two agents and, to a greater extent, the combined treatment with panobinostat and HNE induced G2/M arrest. Furthermore, the combination of panobinostat and HNE induced significant DNA damage, concomitant with the mitotic arrest [106]. Similarly, a G2/M cell cycle arrest accompanied by DNA damage has been reported in hepatocellular carcinoma HepG2 (*p53 wild type*) and Hep3B (*p53 null*) cells treated with HNE [107]. The mechanism involved in apoptosis induction has been deeply investigated. Ji et al. demonstrated that HNE treatment induced cell death in MG63 human osteosarcoma cells, through the activation of caspase-3, due to the inhibition of the activity of AKT and of the downstream factors p70S6K [108]. In prostate cancer cells, HNE promoted apoptosis through the p53 signaling pathway [109]. Moreover, it has been recently reported that HNE induced apoptosis in a wide variety of tumor cells expressing NADPH oxidase 1 (NOX1), a ROS-producing enzyme, by inactivating their membrane-associated catalase. HNE appeared to act by reactivating subsequent intercellular signaling through the NO/peroxynitrite and HOCl pathways, followed by the activation of the mitochondrial pathway of apoptosis [110].

In addition to these specific effects for the different types of cancer cells, it has to be considered that LPO-derived aldehydes are able to form adducts with DNA in cancer cells. This characteristic makes their action similar to the action of some chemotherapeutic drugs, such as cisplatin or doxorubicin, which act by altering DNA structure and interfering with DNA repair mechanisms. Indeed, cisplatin acts by: (1) covalently binding to the purine bases in DNA, thus causing DNA damage, and (2) interfering with DNA repair mechanisms, an effect which is followed by the induction of apoptosis [111]. Doxorubicin binds to nucleic acids, presumably by specific intercalation of the planar anthracycline nucleus within the DNA double helix, and thus poisons topoisomerase II. In this way, doxorubicin interferes with DNA repair and induces apoptosis [112]. Moreover, both these drugs determine increases of oxidative stress, which can contribute to DNA damage and LPO induction. The latter, in turn, can cause increases in the production of reactive aldehydes, thus enhancing the toxic effect of the drugs [113].

Another similarity between chemotherapeutic drugs and HNE is highlighted by the observation that cells can develop resistance after continuous exposure to both kinds of compounds. Indeed, Cipak et al. reported that a strain of yeast, which was able to produce up to 15% peroxidable PUFA, thereby enhancing the production of HNE, was initially more sensitive to oxidative stress than the wild-type strain, but could be rendered more resistant to the stimulation of LPO upon exposure to H_2_O_2_, by increasing the culture timelenght. This adaptation to oxidative stress was linked to an increase in catalase activity [114]. Moreover, it has been demonstrated that HNE production plays a key role in the adaptation to stress [115]. Analogously, cancer cells, particularly in advanced stages of the neoplastic disease, become highly adapted to intrinsic or drug-induced oxidative stress, by up-regulating their antioxidant systems [116]. This occurs via the activation of redox-sensitive transcription factors, such as nuclear factor kappa B (NF-κB) and Nrf2, causing increased expression of ROS-scavenging enzymes and compounds, such as superoxide dismutase and glutathione, elevation of survival factors, such as apoptosis regulator Bcl-2 (BCL2) and induced myeloid leukemia cell differentiation protein Mcl-1 (MCL1), and inhibition of cell death effectors, such as caspases [117],[118]. Thus, in order to overcome the drug resistance associated with redox adaptation, it is important to design strategies which may disable redox adaptation mechanisms in cancer cells. It was shown that the depletion of GSH, obtained by the use of the natural compound beta-phenylethyl isothiocyanate (PEITC), could preferentially kill Ras-transformed ovarian cells [119]. The same result was obtained in leukemic cells from patients with fludarabine-resistant chronic lymphocytic leukemia, in which the use of PEITC effectively eliminated the drug-resistant cell populations [120],[121].

### 4.3. LPO-derived aldehydes and their adducts with DNA as cancer biomarkers

The different rates of production, metabolism and extrusion make the concentrations of LPO-derived aldehydes and their adducts very variable in tumor cells. In patients with different types of kidney tumors, HNE-protein adducts were detected both in normal and tumor cells, although immunomorphologic analyses revealed smaller amounts of HNE-protein adducts in tumor cells [122]. *In vivo* studies on human colon adenocarcinomas at different TNM stages and grades showed that the concentration of HNE was lower in cancer colon biopsies, with respect to normal surrounding tissues [99]. On the contrary, other experimental results indicated that the concentrations of malondialdehyde and HNE were increased in colorectal cancer tissues [123]. In thyroid tumors with a high level of oxidative stress, the contents of HNE and of the DNA lesion 8-OHdG were significantly higher than in normal tissue [87]. Analogously, increased LPO seemed to be a common pathological aspect in astrocytic and ependymal glial tumors, in which the incidence of HNE-immunopositive tumor cells increased with increasing grades of malignancy [124]. In breast cancers at different degrees of malignancy, 8-OHdG levels were diminished significantly in invasive breast carcinomas, compared to non-invasive lesions; conversely, HNE immunostaining was strongest in invasive breast carcinomas [125]. These contrasting data indicate that HNE concentration is strongly dependent on tumor type and stage. Finally, Peluso et al. found significantly higher levels of M1dG, the MDA adduct with dGuo, in breast fine-needle aspirates from 22 breast cancer patients, compared to 13 healthy controls (mean ratio 5.26), and suggested that increased M1dG formation over normal baseline levels may contribute to breast cancer development [76].

## 5. Aldehydes and DNA damage in inflammation

### 5.1. LPO-derived aldehydes and their adducts promoting and modulating inflammation

It is widely accepted that biologically active aldehydes are produced by membrane LPO, in the course of inflammation, which can accumulate in certain tissues up to concentrations of 10 µM or more [126],[127]. In several experimental models of inflammation, increased concentrations of LPO-derived aldehydes or aldehyde-protein adducts have been demonstrated. It was shown that the hyperproduction of HNE in the adipose tissue of obese patients helped the release of pro-inflammatory cytokines, thus contributing to adipose tissue inflammation [128]. In C57BL/6 mice fed a high-fat diet, body weight gains were associated with increases of HNE-protein adducts in adipose tissue [129]. Increases of LPO-derived aldehydes, acting as cell signal messengers, have been implicated also in atherosclerosis [130]. We have reviewed elsewhere the contribution of LPO-derived aldehydes and oxidized low-density lipoprotein (oxLDL) constituents, including acrolein-lysine, MDA-lysine and HNE-histidine adducts in apolipoproteins B and E, as well as oxidized phosphocholine derivatives and their adducts with proteins, to the pathogenesis of atherosclerosis. These compounds all contributed to endothelial stress, subendothelial monocyte infiltration, endocytosis of oxLDLs by macrophages, conversion of the latter into foam cells, atheroma formation and maturation of vascular-associated DCs [18]. We identified HNE adducts with heat shock 60 kDa protein 1 (HSP60) in human promyelocytic HL-60 and monocytic THP-1 cell lines exposed to HNE *in vitro* [131]. Because HSP60 is well known as a vehicle of the presentation of endogenous peptides to T cells [132] and as a target of autoimmune responses in atherosclerosis [133], we proposed that the modification of HSP60 with HNE may both contribute to the oxidative stress-driven inflammation of arterial intima and act as a switchover to immunity-driven chronic inflammation in atherosclerosis [131]. The proatherogenic effects of HNE have been further reviewed recently [134],[135]. Other inflammation-related diseases associated with the presence of aldehyde-protein adducts are alcoholic liver disorders [136] and chronic alcoholic pancreatitis, in which an increased production of HNE-protein adducts was evidenced in acinar cells adjacent to interlobular connective tissue [137]. We will not discuss further these aspects, as the present review focuses mainly on LPO-dependent modifications of DNA and their implications in human diseases.

Even though most of the literature data, with regard to inflammation, concern the reaction of aldehydes with proteins, it has recently been demonstrated that aldehyde-DNA adducts generated in the course of inflammation play an important role in inducing epigenetic changes which, in turn, can modulate the inflammatory process. Certain oxidation products and adducts of the aldehydes produced by LPO in the course of inflammation with dCyt, dAde and dGuo in DNA can affect the methylation of gene promoters, which are inactive when methylated, but more actively transcribed upon demethylation. Examples include the increase in DNA methylation reported by Turk et al. in association with the formation of 8-OHdG [138], and the perturbation of DNA methylation associated with the formation of 3,*N4*-etheno-5-methyl-2′-deoxycytidine which was observed by Nair et al. [139]. Moreover, by exposing Caco-2/15 cells to the iron-ascorbate oxygen radical-generating system, Yara et al. found that the up-regulation of the inflammatory process, as revealed by the activation of NF-κB, was accompanied by increases of LPO and MDA concentration. Interestingly, the assessment of the promoter methylation status revealed decreased levels for the superoxide dismutase 2 (*SOD2*) gene and increased levels for the glutathione peroxidase 2 (*GPX2*) gene, which could be reversed by the treatment with antioxidants, suggesting that LPO products might be implicated in the epigenetic modifications cited [140].

### 5.2. Adducts of LPO-derived aldehydes with DNA as biomarkers of cancer-prone inflammatory diseases

It was suggested that promutagenic etheno-DNA adducts generated in the course of chronic inflammation might act as a driving force for malignant transformation in cancer-prone inflammatory diseases. Increased levels of 1,*N*6-etheno-deoxyadenosine (ε-dAde) and 3,*N*4-etheno-deoxycytidine (ε-dCyt) were detected in the inflamed pancreatic tissues of patients with chronic pancreatitis, in comparison with non-inflamed tissues, and increased levels of ε-dCyt were detected also in the affected colonic mucosa of patients with Crohn's disease and ulcerative colitis [141]. Moreover, highly increased levels of ε-dAde were excreted in the urine of patients with inflammatory cancer-prone liver diseases (chronic hepatitis, liver cirrhosis), as well as hepatocellular carcinoma (HCC) caused by HBV or HCV infection or alcohol abuse. On this base, it was suggested that massive LPO-mediated DNA damage *in vivo* might contribute to the development of HCC. Moreover, it was proposed that ε-dAde levels in urine and target tissues should be further explored as a putative risk marker for the malignant progression of inflammatory liver diseases and may serve as biomarkers to assess the efficacy of preventive and therapeutic interventions [142].

## 6. Adducts of reactive aldehydes with DNA and proteins in autoimmunity

The formation of the adducts of reactive aldehydes produced by LPO with DNA and proteins deeply affects both innate and adaptive immune responses, possibly triggering autoimmunity [18],[143],[144]. Various oxidation-specific epitopes (OSEs) are recognized as endogenous damage-associate molecular patterns (DAMPs) by innate pattern recognition receptors (PRRs). Moreover, the covalent modification of DNA and proteins with LPO products might result in the alteration of self antigens and the generation of neo-epitopes which, in turn, might be instrumental in overcoming the immunological tolerance of autoreactive T and B cells towards self antigens. In fact, it was consistently reported that the modification of macromolecular self antigens with reactive aldehydes not only incited immunological responses to modified antigens, but was accompanied also by the breaking of immunological tolerance to their native counterparts, as discussed below. The latter effect seems to entail the intramolecular spreading of immunological responses from aldehyde-modified to other unmodified epitopes of the same macromolecular antigens and may be viewed as a reflection of the multivalent character of the latter, i.e., the presence within them of multiple antigenic determinants. Moreover, the intermolecular epitope spreading between aldehyde-modified protein antigens and other proteins and/or DNA, either in native or in aldehyde-modified form, was also observed. Such an effect, which appears to be a reflection of the pleiotropic ability of aldehydes to react with many different targets, might result both from the cross-reactivity to aldehydes as shared epitopes in multiple antigens and the molecular mimicry between aldehyde-containing and structurally related self antigenic determinants. The following paragraphs provide an overview of the studies supporting these mechanisms.

### 6.1. Adducts of LPO-derived aldehydes with biological macromolecules as damage-associated molecular patterns

OSEs recognized as DAMPs by PRRs include the oxidation products of membrane phospholipids and PUFAs in LDLs and their adducts. The PRRs involved include scavenger receptors CD36 and SR-B1, Toll-like receptors, C-reactive protein and complement factor H [145]. The adducts of MDA and HNE with LDLs are recognized also by the the so-called “natural” IgM antibodies detected in the sera of immunodeficient *rag1-/-* mice after reconstitution with B-1 cells [146]. The recognition of HNE-modified self antigens, i.e., HNE-histidine adducts in oxLDLs as tissue damage signals, upon the binding of oxLDL to human lectin-like, oxidized low-density lipoprotein receptor 1 (LOX-1) [147] at the surface of DCs, resulted in: a) the activation of DCs, i.e., the upregulation of scavenger receptors and b) the maturation of the DC's presenting capabilities, associated with the enhanced expression of costimulatory molecules [148].

### 6.2. Aldehyde adducts inciting autoimmunity via modification of self antigens and intramolecular epitope spreading

Numerous observations indicate that the modification of self antigens with the products of LPO might favour the breaking of immunological tolerance. Murine serum albumin (MSA), modified *in vitro* with several unsaturated (MDA, HNE, heptadienal) and saturated aldehydes (butanal, nonanal), induced strong T-cell-dependent antibody responses, unlike native MSA. Even though certain T-cell hybridomas established from immunized mice recognized MDA- and HNE-modified MSA, but not native MSA, the sensitization of T cells to HNE-MSA favoured the intramolecular spreading of the immune response to formerly tolerated epitopes of the native self antigen. In fact, HNE-MSA and nonanal-MSA induced antibody responses to unmodified MSA almost as intense as to aldehyde-modified MSA [149]. Similarly, an autoimmune response to the SS-A2/Ro60 antigen was established faster and more strongly in rabbits immunized with HNE-modified SS-A2/Ro60, as compared with the native antigen [150],[151]. The SS-A1/Ro52, SS-A2/Ro60 and SS-B/La antigens are the targets of certain antinuclear autoantibodies (ANA) characteristically detected in Sjögren syndrome (SS) and systemic lupus erythematosus (SLE) [154]. SS is an immunity-driven chronic inflammatory disorder, characterized by keratoconjunctivitis with dry eyes and xerostomia, caused by the infiltration and destruction of lacrimal and salivary glands by effector CD4^+^ and CD8^+^ T cells and activated macrophages. It has a prevalence of 1%, with affected females outnumbering males by 9:1. SLE is a multisystemic disease marked by a polyclonal B cell activation, with plasma cells producing ANA towards a broad range of autoantigens, including double stranded DNA, histones, and a number of ribonucleoprotein particles (RNPs), such as the Smith antigen (i.e., the common core proteins of spliceosomal small nuclear RNPs), and the SS-A/Ro and SS-B/La antigens. Immune complexes and complement depositing in the wall of small arteries, at the dermo-epithelial junction and in the glomerular basal membrane are responsible, respectively, for the vasculitis, the erythematous, bullous and ulcerative skin lesions and the nephritis accompanying SLE. SS-A2/Ro60 (TROVE2) is involved in cell responses to UV damage; SS-A1/Ro52 (TRIM21) is a E3 ubiquitin-protein ligase involved in the regulation of innate immunity, inflammation in response to IFN-γ, and autophagy; the 48-kDa SS-B/La antigen, instead, is a transcription termination factor for RNA polymerase III. All three are components of RNPs, in which they are associated with short, non-coding, histidine-rich RNAs (HY-RNAs). Autoantibodies to SS-A2/Ro60 are found in more than 60% of SS patients and 25–40% of SLE patients, as well as in patients affected by other autoimmune diseases [152]. SS-Ro and SS-La antigens become exposed in apoptotic bodies and blebs at the surface of apoptotic cells [153], where they are accessible to the binding by autoantibodies [154]. It was suggested [155] that, in SS and SLE, the triggering of autoimmunity might be favoured both by an intrinsic susceptibility of leukocytes to apoptosis [156]–[159], possibly due to the overexpression of the E3 ubiquitin ligase SS-A1/Ro52 [160], and by an impaired efferocytosis of apoptotic cells by macrophages [158]–[161]. The presentation of self antigens by thymocytes was shown to be enhanced in late apoptosis, but the production of autoantibodies was shown to require the modification of self antigens, with the formation of neo-epitopes [162]. Therefore, it was proposed that LPO-mediated oxidative modifications occurring as a consequence of the enhanced oxidative stress that accompanies apoptosis might help overcome the immunological tolerance to self antigens [153].

Furthermore, Kurien et al. reported that anti-SS-A2/Ro60 and anti-SS-B/La antibodies were produced in response to HNE-modified SS-A2/Ro60. The response was faster and stronger when the immunogen had been modified with 0.4 mM and even more so with 2 mM HNE, in which case anti-double-strand DNA (dsDNA) antibodies also were formed. The antibodies produced by mice immunized with HNE-modified, but not with unmodified SS-A2/Ro60, included added subpopulations which recognized HNE or HNE-SS-A2/Ro60 and dsDNA, but not the native antigen [163]. The occurrence of anti-SS-B/La and anti-dsDNA antibodies, after immunization with unmodified or HNE-modified SS-A2/Ro60, illustrates the concept of intermolecular epitope spreading, which will be discussed further below. The immunogenicity of human serum albumin (HSA) in female NZW rabbits was also markedly enhanced by HSA modification with HNE and appeared to entail the extension of the antibody response to DNA. Although anti-HNE-HSA antibodies were highly specific for the immunogen, they also recognized unmodified HSA, suggesting that sensitization to HNE-dependent epitopes was accompanied by intramolecular spreading to shared native HSA epitopes [164]. They also exhibited cross-reactivities with HNE-modified forms of bovine serum albumin (BSA), N-acetyl-l-lysine, N-acetylhistidine, cysteine and - most relevant for the present discussion - native and HNE-modified calf thymus DNA [165]. However, serum antibodies from 27 of 40 patients with SLE preferentially bound to HNE-HSA, with respect to DNA and native HSA [164].

The mechanism by which the formation of aldehyde adducts might promote immunological responses to formerly tolerated self antigens likely reflects a combination of the effects of LPO products on antigen-presenting cells (APCs) and of the formation of neo-epitopes by the modification of self antigens. As already mentioned, the recognition of HNE-containing neo-epitopes, such as HNE-histidine adducts in oxLDLs, by PRRs produced two effects in DCs: (1) the upregulation of scavenger receptors, such as LOX-1, facilitating the uptake of HNE-modified antigens, and (2) the enhanced expression of costimulatory molecules, permitting the sensitization of neo-epitope-recognizing CD4^+^ T cells [148]. Neo-epitope-recognizing CD4^+^ T cells are selected outside the repertoire of autoreactive T cells, which were either clonally deleted or put under regulatory control during T cell development. Once sensitized, neo-epitope-specific effector Th2 cells might cooperate with neo-epitope-specific B cells, recognizing HNE-related neo-epitopes with their BCRs, so to induce their differentiation into memory B cells and plasma cells producing neo-epitope-specific antibodies. It may be worth considering that, due to the multivalent character of protein antigens, B cells internalizing HNE-modified proteins via BCRs which recognize native self epitopes actually are able to present two kinds of antigenic determinants to T cells simultaneously: (1) unmodified self epitopes to autoreactive, resting CD4^+^ T cells; (2) HNE-containing epitopes to neo-epitope-specific, effector Th2 cells. In this way, autoreactive B cells might gain access to the cooperation provided by neo-epitope-specific Th2 cells and might differentiate into autoantibody-producing plasma cells and memory B cells. To the same extent, APCs which uptake and process HNE-modified proteins might present at the same time: (3) HNE-containing neo-epitopes to neo-epitope-specific CD4^+^ T cells; (4) unmodified self epitopes to autoreactive, resting CD4^+^ T cells. Added stimulation to express costimulatory signals might come to these APCs from OSEs binding to PRRs and from CD4^+^ T cells recognizing HNE-containing neo-epitopes, helping them overcome the immunological tolerance of autoreactive, resting CD4^+^ T cells. The latter might thus differentiate into autoreactive effector Th2 cells, which, in turn, might promote the differentiation of autoantibody-producing plasma cells [18],[144].

### 6.3. Aldehyde-containing neo-epitopes as shared or structurally related determinants, favoring the intermolecular spreading of autoimmunity between proteins and from proteins to DNA

The ability of HNE to form adducts with multiple macromolecules, i.e., a broad array of conjugates sharing the HNE moiety as a common epitope, might partly explain the wide range of autoantibody responses occurring in SLE and SS. The latter might rely on crossed reactions, partly based upon the sharing of the HNE group as a common antigenic determinant and partly on the epitopic mimicry between HNE-containing and structurally related epitopes. The HNE moiety was recognized as the common antigenic determinant targeted by the antibodies raised against a number of HNE-protein adducts. Anti-HNE-LDL antibodies raised in rabbits recognized also HNE-albumin and HNE-high-density lipoprotein 3 (HDL3), in addition to HNE-LDL, but non MDA-LDL, which indicated antibody specificity towards HNE-containing epitopes, irrespective of the carrier protein [166]. Furthermore, HNE-specific antibodies raised in NZW rabbits against a HNE-keyhole limpet hemocyanine (KLH) conjugate recognized glyceraldehyde-3-phosphate-dehydrogenase (GAPDH) modified with HNE in an immunoblot assay, with an intensity which was proportional to the number of HNE-histidine adducts in GAPDH. Antibody binding was fully inhibited by HNE-acetyl-l-lysine, HNE-N-acetylhistidine and HNE-glutathione [167].

**Figure 9.**
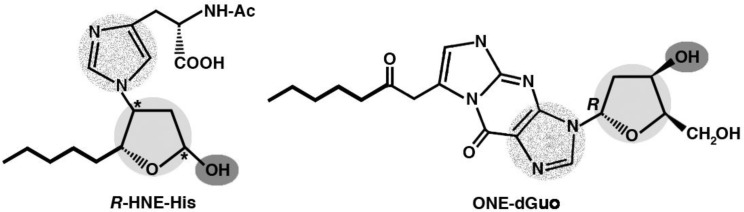
Molecular mimicry between the *R*-HNE-histidine and the 7-(2-oxo-heptyl)-1,*N*2-etheno-type ONE-2′-deoxyguanosine (ONE-2′-dGuo) adducts. Shared or closely resembling functional groups implicated as the components of a shared epitope, responsible for the molecular mimicry between the two adducts and required for the recognition by bispecific antibodies, are highlighted by shades of *grey*. Color-code: *light grey*, 2′-deoxyribose-like tetrahydrofuran rings; *dark grey*, hydroxyl groups; *dotted grey*, nitrogen-containing heterocyclic groups (histidine and guanine). The shared pentyl groups of the HNE-histidine and ONE-2′-deoxynucleoside adducts (indicated by bold lines) are probably also involved in the recognition by antibodies.

Interesting studies were conducted on the molecular mimicry between proteins modified with HNE and its analogs and DNA, in native or modified form, as a possible mechanism for the appearance of anti-DNA autoantibodies in response to HNE-modified self protein antigens. The sequence determined for an anti-HNE monoclonal antibody (anti-*R* mAb 310), selectively recognizing the *R* enantiomer of HNE-histidine Michael adducts [168], strictly resembled the sequences of various clonally related anti-DNA antibodies. Despite this similarity, the cross-reactivity of mAb R310 with native dsDNA was limited, but strongly enhanced by the treatment of DNA with 4-oxo-2-nonenal (ONE) (Figure 1). Within the context of ONE-modified DNA, ONE-2′-deoxynucleoside adducts were identified as alternative epitopes of mAb R310. The constituent chemical groups of a common epitope, possibly responsible for the molecular mimicry between the *R*-HNE-histidine and the 1,*N*2-etheno-type ONE-2′-deoxyguanosine adducts, and required for the recognition by bispecific antibodies, were delineated (Figure 9). It was proposed that endogenous electrophilic molecular species like HNE may trigger autoimmune disease [169].

**Figure 10.**
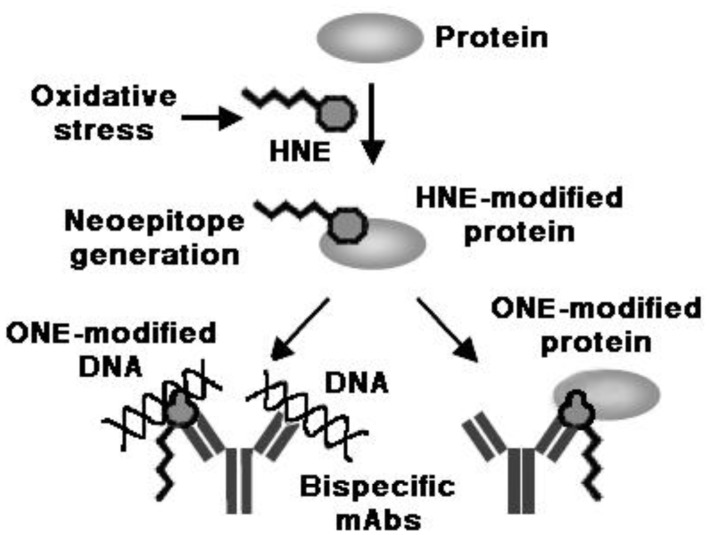
HNE-specific epitopes as endogenous triggers of anti-DNA antibody responses. Immunization with HNE-modified KLH resulted in the production of monoclonal bispecific antibodies recognizing both native and ONE-modified DNA, on one hand, and ONE-modified BSA, on the other hand [170]. mAbs, monoclonal antibodies.

Furthermore, upon repeated immunization of mice with HNE-modified KLH, a distinct population developed of B cell clones, which recognized native DNA, and, to a greater extent, ONE-modifed DNA, but not HNE-BSA. Anti-DNA mAbs, in turn, cross-reacted with ONE-modified BSA. It was suggested that HNE-specific epitopes of HNE-modified proteins might act as sensitizing antigenic determinants for the production of bispecific antibodies recognizing native and ONE-modified DNA, on one hand, and ONE-modified proteins, on the other hand [170] (Figure 10). Moreover, it was reported that IgG antibodies, raised in rabbits against HNE-modified HSA, recognized HSA from SLE patients and cross-reacted with native and oxidized goat liver chromatin. In turn, anti-native/oxidized chromatin antibodies from 41 of 74 SLE patients specifically recognized HNE-HSA [171].

On the whole, the findings reported above strongly support the pathogenic role of the adducts formed by the products of LPO with biological macromolecules in the breaking of immunological tolerance to self antigens and in autoimmunity. However, the instrumental role of the adducts of reactive LPO products with self protein antigens in the sensitization to the corresponding unmodified proteins and in the intermolecular spreading of the autoimmune responses to aldehyde-modified and native DNA seems better documented than the reverse flow of immunological activation from aldehyde-modified DNA to protein antigens. Investigation in the occurrence and circumstances of the immunological responses to the adducts of DNA with the products of LPO and in their possible role in the spreading of the immunological response to similarly modified, unmodified or structurally analogous self protein antigens is warranted. Table 1 contains a list of commonly hypothesized mechanisms for breaking immunological tolerance to self antigens, in the context both of infection and of oxidative stress, emphasizing the possible role of the adducts of LPO-derived reactive compounds.

**Table 1.** Possible mechanisms for breaking immunological tolerance to self antigens, underlining the role of: (a) infection; (b) the formation of adducts of LPO-derived aldehydes with self antigens.

General mechanism	mechanism according to disease context	
	(a) infection	(b) oxidative stress/LPO
APC activation	recognition of PAMPs by APCs presenting self antigens	recognition of DAMPs/OSEs by APCs presenting self antigens
Modification of self/neo-epitope formation	binding of microbial components to self antigens	oxidative modification of self antigens, including adduct formation with LPO products
Intramolecular spreading	activation of T helper function ^(1)^ by the microbial component of microbial-self antigen complexes	activation of T helper function ^(2)^ by the LPO-derived component of aldehyde adducts with self antigens
Intermolecular spreading: a) by epitope mimicry	molecular mimicry between microbial and structurally related self epitopes	molecular mimicry between aldehyde-modified and structurally related self epitopes
Intermolecular spreading: b) by cross-reaction	cross-reactivity to microbial components as shared antigenic determinants in multiple antigens	cross-reactivity to LPO-derived components as shared antigenic determinants in multiple antigens
Bystander activation	cytokine-mediated activation of autoreactive T cells during a response to microbial antigens	cytokine-mediated activation of autoreactive CD4^+^ T cells during a response to LPO-modified antigens
Destruction of anatomical barriers	permitting naïve T and B cells to access segregated antigens within immunologically privileged sites	
Exposure to superantigens	polyclonal activation of autoreactive T and B cells	

^(1)^ Recruitment of effector Th2 cells sensitized by microbial antigenic determinants in microbial-self antigen complexes, in aid of: (a) APCs presenting self epitopes from microbial-self antigen complexes to autoreactive, resting CD4^+^ T cells: (b) autoreactive B cells.

^(2)^ Recruitment of effector Th2 cells sensitized by aldehyde-related epitopes of aldehyde adducts with self antigens, in aid of: (a) APCs presenting self epitopes deriving from aldehyde adducts with self antigens to autoreactive, resting CD4^+^ T cells: (b) autoreactive B cells.

### 6.4. Aldehyde adducts with DNA as biomarkers of immunity-driven inflammation and autoimmunity

Non-alcoholic fatty liver disease (NAFLD) spans a spectrum of disease, from relatively benign lipid accumulation (simple steatosis, fatty liver), which is devoid of long-term adverse effects, to progressive non-alcoholic steatohepatitis (NASH), which is associated with necrosis, chronic inflammation and fibrosis, leading to liver cirrhosis. Immunity-driven inflammation seems to be involved in the progression of NAFLD from steatosis to NASH, as hepatic oxidative stress markers, such as HNE and 8-OHdG, correlated with the severity of hepatic necrosis, inflammation and fibrosis [172],[173] and antibody responses to MDA-modified antigens were associated with increased severity of lobular inflammation or fibrosis [174]. Moreover, high levels of the carcinogenic etheno-DNA adduct 1,*N*6-etheno-2′-deoxyadenosine (ε-dAde) were detected in 17 of 21 liver biopsies from young NASH patients, suggesting that LPO-mediated DNA lesions might be implicated in the pathogenesis of NASH. However, whether these adducts may serve as predictive risk markers for the development of hepatocellular carcinoma remains to be investigated [175]. Furthermore, in patients with rheumatoid arthritis (RA), the levels of heptanone-etheno-2′-deoxycytidine, i.e., 7-(2″-oxoheptyl)-3,*N*4-etheno-2′-deoxycytidine (ONE-dCyt) in DNA from whole blood cells were significantly higher and age-dependent, compared with controls, whereas there were no significant differences in 8-oxo-hydroxy-7,8-dihydro-2′-deoxyguanosine (8-oxo-dGuo) and ε-dAde levels. ONE-dCyt levels correlated well with the number of swollen joints and weakly with the number of tender joints of RA patients, indicating that ONE-dCyt may have some influence on RA development [176].

Although we focus here on the adducts of LPO products with DNA, it is worth mentioning that Toyoda et al., while investigating the role of HNE-modified proteins as a possible source of anti-DNA antibodies, detected antibodies against HNE-modified BSA in the sera of patients with SLE, SS, RA, systemic sclerosis and idiopathic inflammatory miopathies, and HNE-specific epitopes in the epidermis and dermis of patients with SLE, pemphigus vulgaris and contact dermatitis [170]. Moreover, Wang et al. reported data which underscore the pathogenic role of LPO in SLE and the potential usefulness of anti-DNA and anti-HNE antibody titers in predicting its progression. Their study showed that the prevalence and serum levels of MDA- and HNE-protein adducts, on one hand, and of MDA- and HNE-specific antibodies, on the other hand, were: (1) interrelated; (2) significantly higher in SLE patients than in healthy controls and (3) in correlation also with the SLE Disease Activity Index [177].

## 7. Conclusions

The interaction of LPO-derived aldehydes with DNA and cellular proteins is undoubtedly at the base of most diseases related to LPO induction. As reported in this review, besides the mutagenic role, a pro-apototic role displayed by aldehyde-DNA adducts in neoplastic cells has been described, as well as the induction of DNA methylation at specific sites, which can favor the inflammatory process. Moreover, strong support can be found in the scientific literature for the pathogenic role of the aldehyde adducts with biological macromolecules in the breaking of immunological tolerance to self antigens and autoimmunity.

Despite the abundance of studies, the complexities of the reactions between reactive aldehydes and DNA still leave room for further investigation.
